# The in vitro effects of aflatoxin B_1_ on physiological functions of swine alveolar macrophages

**DOI:** 10.1002/vms3.313

**Published:** 2020-06-28

**Authors:** Victor Fei Pang, Chung‐Feng Chiang, Chih‐Cheng Chang

**Affiliations:** ^1^ Graduate Institute of Molecular and Comparative Pathobiology School of Veterinary Medicine National Taiwan University Taipei Taiwan; ^2^ Kuo Hsing Poultry and Livestock Feeds Co., LTD. Pingtung Taiwan; ^3^ Department of Veterinary Medicine College of Veterinary Medicine National Chiayi University Chiayi Taiwan

**Keywords:** aflatoxin, swine alveolar macrophage, toxicity

## Abstract

The toxic effects of aflatoxin B_1_ (AFB_1_) on the physiological functions of swine alveolar macrophages (SAM) were investigated. Freshly isolated SAM were incubated with various AFB_1_ concentrations (1.6 × 10^–1^ – 1.6 × 10^5^ nmol/L) and time periods, and their phagocytic ability, synthesis of DNA, RNA and protein, and cell activation by lipopolysaccharide (LPS), were analysed. Results demonstrated that a significant (*p* < .05) reduction (60%) in *Staphylococcus aureus* uptaken by SAM appeared 3 hr after AFB_1_ (>16 nmol/L) treatment. The synthesis of DNA, RNA and protein were markedly reduced, among which DNA and protein synthesis were affected more noticeably. The activation of SAM by LPS was significantly (*p* < .05) suppressed when the concentration of AFB_1_ reached 1.6 × 10^3^ nmol/L. In general, most of the analysed effects were more prominent as AFB_1_ concentration or incubation period increased. Taken together, AFB _1_could elicit significant adverse effects on the physiological functions of SAM. Exposure of pigs to aflatoxin‐contaminated feed may increase their susceptibility to various secondary infections.

## 1 INTRODUCTION

Mycotoxin contamination is always a serious threat in human and animal health. The impact on animal health involves reduced reproduction, immunity and production efficiency, so that susceptibility to disease and cost associated preventing mycotoxin contamination are increased. Aflatoxin (AF) is one of the mycotoxins commonly contaminated in animal feeds worldwide (Broom, 2015; Bryden, 2012). Pigs exposed to feed with various levels of AF contamination could develop acute death in severe cases, and chronic mycotoxicoses. The consequences of this chronic effect in pigs include hepatoxic damages, complexity in physiological functions and finally increased susceptibility to diseases (Bryden, 2012; Pierron, Alassane‐Kpembi, & Oswald, 2016; Wild & Gong, 2010). It has been indicated that this toxin could increase the susceptibility of pigs to pulmonary pathogens, and further exacerbate porcine respiratory disease (Park, Kim, Kim, & &Moon, 2015), one of the most pervasive diseases in the pig industry.

AFs are a group of bifuranocoumarin metabolites produced mainly by *Aspergillus flavus* and *A. parasiticus* (Bryden, 2012; Creppy, 2002; Panangala et al., 1986; Pierron et al., 2016; Streit, Naehrer, Rodrigues, & Schatzmayr, 2013). Among which, AFB_1_ is the most potent and commonly produced toxin (Panangala et al., 1986; Streit et al., 2013). In addition to the acute death and chronic hepatocarcinogenicity, immunosuppression has long been recognized as one of the adverse effects associated with aflatoxicosis (Meissonnier et al., 2008; Pierron et al., 2016). It has been reported that AFs can impair various non‐specific and specific humoral and cellular immunities as well as disease resistance in many animal species (Creppy, 2002; Cysewski, Wood, Pier, & Baetz, 1978; Meissonnier et al., 2008; Panangala et al., 1986; Reddy, Taylor, & Sharma, 1987). Pigs fed with AFB_1_ were found to have delayed cell‐mediated immunity, cell apoptosis, as well as modulation of cytokine expression, that is affecting the synthesis of functional proteins (Mehrzad, Bahari, RezaBassami, Mahmoudi, & Dehghani, 2018; Meissonnier et al., 2008; Qian et al., 2014).

Macrophages play an important role in the physiological defence system, including phagocytosis, antigen procession and presentation, cytokine production as well as pathogen and tumour cell destruction (Varol, Mildner, & Jung, 2015). Thus, any factor interfering with macrophage functions may result in the reduction in immunity and immunological functions. Consumption of AF‐contaminated feed reduced the clearing ability, phagocytic ability and cytokine secretion of T‐cell subsets and macrophage lineage cells in broiler chickens, rabbit and mice (Chang & Hamilton, 1979; Dugyala & Sharma, 1996; Kadian, Monga, & Goel, 1988; Liu, Jiang, Fang, Peng, & Cui, 2016; Michael, Thaxon, & Hamilton, 1973; Richard & Thurston, 1975), Following incubation with AFB_1_ in vitro, there was a reduction in substrate adherence potential and phagocytic activity in chicken peritoneal macrophages (Neldon‐Oritz & Qureshi, 1992).

The physiological function of macrophages, such as phagocytosis and production of monokines regulating the functions of both T‐ and B‐cell, depends on the levels of activation via exterior stimulation (Meissonnier et al., 2008). It has been observed that activated macrophages preferentially incorporate glucosamine into their cell membrane by means of ultrathin autoradiograph sections and cell fractionation procedures (Hammond & Dvorak, 1972; Reine, Jenssen, & Kolset, 2016). However, many studies related to AFB_1_ were primarily focused on the toxicity of its secondary metabolites, whereas its direct effects on swine macrophages have not been fully elucidated (Cortinovis, Pizzo, Spicer, & Caloni, 2013; Meissonnier et al., 2008). In this study, the effects of AFB_1_ on the functions of swine alveolar macrophages (SAM) were evaluated. Parameters analysed in AFB1‐treated SAM were phagocytic ability, DNA, RNA and protein synthesis, as well as cell activation by lipopolysaccharide (LPS) in vitro.

## 2 MATERIALS AND METHODS

### 2.1 Animals

Totally, three 8‐ to 10‐week‐old Yorkshire X Landrace crossbred pigs (females or castrated males) were used for obtaining SAM. All procedures involving animal handling and treatments were adhered to the spirit of Animal Welfare Act legislated by Legislative Yuan, Republic of China (Taiwan). The authors also confirm that the ethical policies for animal welfare, appeared on the journal's author guidelines pages, particular the handling guidelines were followed. Also, the ethical issues, that is appropriate protocols of humane anaesthesia, involved in this study were carefully executed and adhered to the essence of EU standards for the protection of animals used for scientific purposes.

### 2.2 Preparation of toxin

Pure AFB_1_ (Serva, Heidelberg, FRG) was resolved in 100% dimethyl sulphoxide (DMSO) to prepare a stock AFB_1_ solution at a concentration of 3.2 × 10^5^ nmol/L that was stored at −20°C. AFB_1_ working solutions with concentrations ranging from 3.2 × 10^–1^ to 3.2 × 10^5^ nmol/L were prepared by 10‐fold serial dilutions with RPMI 1,640. Parallel cultures, including DMSO and medium control, were also set up. The final AFB_1_ and DMSO control testing concentrations were 1.6 × 10^–1^ to 1.6 × 10^5^ nmol/L and 0.5%, respectively, obtained by mixing equal volumes of suspended SAM and AFB_1_ or DMSO control working solutions.

### 2.3 Bronchoalveolar lavage, cell viability and differential cell count

The trachea and lungs were collected immediately after the pigs were humane anesthetized by intravenous injection of thiamylal sodium cytosol (5mg/kg body weight), followed by bled and necropsied. Bronchoalveolar lavage was performed with a technique modified from an earlier research (Senior, Edward, Campbell, & Villiger, 1981). Briefly, 50–100 ml of cold, sterile calcium and magnesium‐free Dulbecco's PBS (D‐PBS) supplemented with 0.2% ethylenediaminetetraacetic acid (EDTA) were infused to the trachea and then flowed into the lung. Following a generalized gentle massage, the fluid was then poured through a layer of gauze (to remove mucus) into a sterile siliconized bottle. The procedure was repeated several times until a total of 1 litre of D‐PBS was used. The recovery lavage fluid was centrifuged at 400 *g* for 10 min at 4°C. The cell pellet was then resuspended in 10 ml of 0.83% ammonium chloride RBC‐lysing medium for 5 min. Then 40 ml of RPMI 1,640 without foetal bovine serum was added and the cells were centrifuged again at 320 *g* for 10 min at 4°C. Following two more washes the cells were resuspended in 10 ml of RPMI 1,640 culture medium which had been supplemented with heat‐inactivated foetal bovine serum (HIFBS)(10%), l‐glutamine (2 nM) and penicillin (100 units/ml) and streptomycin (100 µg/ml)(P/S). The percentages of viable SAM and non‐viable SAM were determined by light microscopy on the basis of Trypan blue exclusion followed by dividing the number of viable cells by the total number of cells. The cell suspension was then further diluted and adjusted to a final concentration of 2 × 10^7^ or 5 × 10^6^ live SAM/ml in RPMI 1,640 culture medium.

### 2.4 Phagocytosis assay

A live virulent *Staphylococcus aureus* strain (CCRC 10,779) was used in the phagocytosis assay. The bacteria were opsonized by incubation with heat‐inactivated pooled normal swine serum for 30 min at 37°C. Thereafter, 2 ml of *S. aureus* culture (4 × 10^8^ bacteria/ml) was mixed with an equal volume of SAM suspension (bacteria: SAM = 20:1) which was pretreated with AFB_1_ for 3, 6 and 9 hr, respectively, at 37°C. The cell‐bacteria suspension was incubated on a rotary shaker for different periods (30, 60, 90, or 120 min) and the phagocytic ability of SAM was determined. Approximately 50 μl aliquots of the cell‐bacteria suspension were used for making cytospin smears (80 *g* for 10 min) stained by Diff Quick staining solution (American Scientific Products). The phagocytic ability of SAM was expressed as the phagocytic index (percent SAM containing bacteria), which was obtained by counting the number of SAM containing 1 or more bacteria in 200 randomly selected cells under a light microscopy. The data were expressed as the delta (Δ) phagocytic index, where phagocytic index = phagocytic index for the AFB_1_‐treated group − phagocytic index for the DMSO control group (Pang et al., 1987).

### 2.5 DNA, RNA and protein synthesis

Aliquots of SAM suspension were added to the wells of 12‐well culture plates to have the cell density equal to 5 × 10^5^ cells/cm^2^. The SAM monolayers were treated with various concentrations of AFB_1_ as described earlier and incubated with regular RPMI 1,640 containing 2 μCi/ml [^3^H‐methyl]‐thymidine ([^3^H]TdrR) (sp. act. 6.7 mCi/mM; NEN) (Tanaka, Nagao, Imai, & Mori, 1980; Gerberick, Sorenson, & Lewis, 1984) and 2 μCi/ml [glucosamine‐6‐^3^H(N)]‐uridine (sp. act. 9.1 Ci/mM; NEN) for subsequent analysis of DNA and RNA, respectively. At the end of incubation period (24 or 48 hr), the cells were rinsed with PBS containing 10 μM cold uridine; solubilized suspensions were then placed in scintillation vials. Following the addition of 10 ml of scintillation fluid (Hydrofluro; National Diagnostics), the radioactivity was counted (Gerberick et al., 1984). For protein synthesis, SAM monolayers were incubated in leucine‐free RPMI 1,640 (Gibco Laboratories) supplemented with 10% HIFBS, 2 mM L‐glutamine and P/S. The SAM monolayers were incubated with AFB_1_ of various concentrations and 1 μCi/ml L‐[3,4,5‐^3^H(N)]‐leucine (sp. act. 153 Ci/mM; NEN) at 37°C in 5% CO_2_ for 24 or 48 hr (Gerberick et al., 1984). At each time point, the cultured medium was discarded and the cells were washed twice with PBS. The monolayers were solubilized with 0.5 ml of 7 M guanidine‐HCl, acidified with 2 ml of 10% trichloacetic acid (TCA). One hundred and fifty microlitre of 1% bovine serum albumin was then added. The precipitate was collected and placed in scintillation vials by plastic disposable paste pipettes that were covered by a piece of Scotties facial tissue. Following the addition of 10 ml of scintillation fluid, the radioactivity was counted in a scintillation counter (LS 6,000 IC; Beckman Inst. Inc.). Results were expressed as difference in counts per minute (DCPM), where DCPM = (average CPM of 3 AFB_1_‐treated cultures of a particular concentration) − (average CPM of 3 DMSO cultures of the same concentration).

### 2.6 Activation of SAM by lipopolysaccharide

SAM monolayers were prepared as described earlier and incubated in 1 ml of regular RPMI 1,640 supplemented with 10% HIFBS, P/S, and L‐glutamine and containing 10 μg/ml LPS (*E. coli* 055:B5) (Sigma Chemical Company) for 15 hr at 37°C in 5% CO_2_ (Gerberick et al., 1984). Control cultures received no LPS. After 15 hr of incubation, the medium was aspirated. One millilitre of the culture medium containing 10 μg/ml LPS and 1 μCi/ml D‐[1, 6‐^3^H(N)]‐glucosamine (sp. act. 60 Ci/mM; NEN) and an equal volume of AFB_1_ of various concentrations were added to each well of 12‐well plate and incubated for 24 or 48 hr. The monolayers were then rinsed with PBS and solubilized with 250 μl of 3% Triton X‐100 for 20 min. The solubilized suspensions were placed in scintillation vials. Following the addition of 10 ml scintillation fluid, the radioactivity was counted. Results were expressed as difference in counts per minute (DCPM), where DCPM = (average CPM of 3 AFB_1_‐treated cultures of a particular concentration) − (average CPM of 3 DMSO cultures of the same concentration).

### 2.7 Statistical analysis

All the data were subjected to an analysis of variance using the GLM (General Linear Model) procedure of the Statistical Analysis System in which the F‐ratios were calculated. If a significant F‐ratio was obtained, the significant differences among treatments were then further calculated by Duncan's multiple range test. The *p* values <.05 and .005 were significantly and very significantly different.

## 3 RESULTS

**Figure 1:**
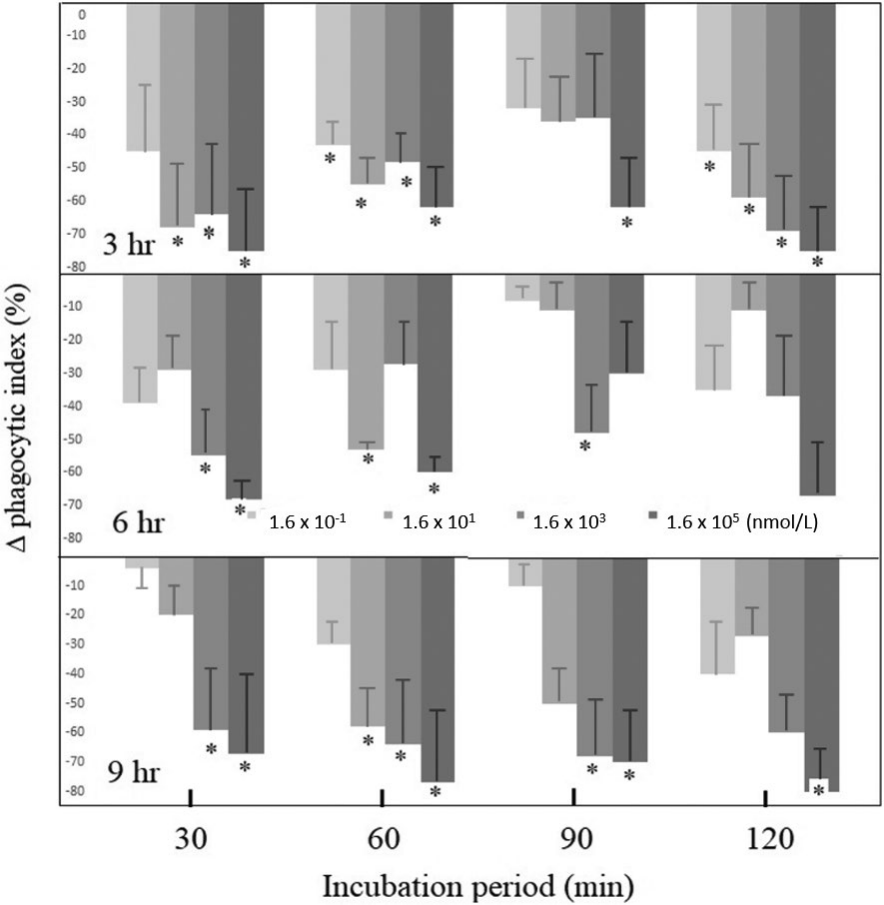
The SAMs were treated with different concentrations of AFB1 (light to dark the delta (Δ) = AFB1‐treated group ‐ phagocytic index for the DMSO control group. * Indicates the significant difference (*p* < 0.05).

The phagocytic indices of the AFB_1_‐treated SAM were consistently lower, ranged 5 to 90%, than those of SAM treated with DMSO alone (the DMSO controls) (Figure 1). The phagocytic ability of SAM to uptake *S. aureus* was significantly (*p* < .05) reduced by more than 60% when SAM were incubated with AFB_1_ at the concentration of 16 nmol/L for only 3hr (Figure 1). There was a tendency that the reduction in the phagocytic ability of AFB_1_‐treated groups was somewhat positively correlated with the AFB_1_ concentrations. However, the time period for AFB_1_ pretreatment (3, 6 and 9 hr) and the time period allowing SAM to perform phagocytosis (30, 60, 90 and 120 min) did not have effects on the phagocytic ability of SAM.

The effects of AFB_1_ treatment on the DNA, RNA and protein synthesis of SAM are illustrated in Tables 1, 2 and 3, respectively. An inhibitory effect was noted in all the three macromolecules tested, in which protein synthesis was the most sensitive to AFB_1_ and RNA synthesis was affected the least. The protein synthesis was significantly (*p* < .05) reduced in SAM incubated with AFB_1_ at a concentration as low as 16 nmol/L for 24 hr (Table 3). The adverse effect became more apparent as the incubation period extended to 48 hr. For DNA synthesis, significantly (*p* < .05) reduced DCPMs were observed in SAM treated with AFB_1_ concentration of 1.6 × 10^1^ nmol/L or above for 24 hr; however, unlike in the protein synthesis, no difference was noted between the concentrations of 1.6 × 10^1^ and 1.6 × 10^3^ nmol/L (Table 1). A significant (*p* < .05) reduction in RNA synthesis was only seen in SAM treated with the highest concentration of AFB_1_ (1.6 × 10^5^ nmol/L) for 24 hr (Table 2). When the treatment of AFB1 was prolonged to 48 hr, the time effect on inhibition was augmented in RNA (1.6 × 10^3^ and 1.6 × 10^5^ nmol/L of AFB_1_), but not DNA synthesis.

**Table 1:** The toxic effects of different concentrations of aflatoxin B_1_ on the DNA synthesis of swine alveolar macrophages tested by radioactivity assay after 24 and 48 hr of incubation in vitro

Conc. of aflatoxin B_1_ (nmol/L)	Incubation period (hr)	
	24	48
DMSO‐C	14,719 ± 1087^a^	14,035 ± 3704^a^
1.6 × 10^–1^	13,330 ± 1448^a^	12,066 ± 2919^ab^
1.6 × 10^1^	11,275 ± 850^b^	10,039 ± 2197^ab^
1.6 × 10^3^	10,069 ± 1171^b^	8,909 ± 2751^b^
1.6 × 10^5^	4,484 ± 597^c^	4,017 ± 1389^c^

Note

Values in the same column with different letters, a, b and c in superscript are statistically different (*p* < .05 and 0.005). The results were expressed as difference in counts per minute (DCPM) and presented as mean ± *SD*.

**Table 2:** The toxic effects of different concentrations of aflatoxin B_1_ on the RNA synthesis of swine alveolar macrophages tested by radioactivity assay after 24 and 48 hr of incubation in vitro

Conc. of aflatoxin B_1_ (nmol/L)	Incubation period (hr)	
	24	48
DMSO‐C	106,131 ± 15,329^a^	109,599 ± 10,758^a^
1.6 × 10^–1^	99,183 ± 15,227^a^	108,948 ± 13,125^a^
1.6 × 10^1^	87,546 ± 14,771^a^	106,050 ± 11,966^a^
1.6 × 10^3^	88,287 ± 16,202^a^	71,529 ± 11,224^b^
1.6 × 10^5^	22,438 ± 5,436^b^	8,141 ± 1693^c^

Note

Values in the same column with different letters, a, b and c in superscript are statistically different (*p* < .05 and 0.005). The results were expressed as difference in counts per minute (DCPM) and presented as mean ± *SD*.

**Table 3:** The toxic effects of different concentrations of aflatoxin B_1_ on the protein synthesis of swine alveolar macrophages tested by radioactivity assay after 24 and 48 hr of incubation in vitro

Conc. of aflatoxin B_1_ (nmol/L)	Incubation period (hr)	
	24	48
DMSO‐C	196,980 ± 29,469^a^	215,261 ± 27,664^a^
1.6 × 10^–1^	165,311 ± 31,332^ab^	224,903 ± 41,074^a^
1.6 × 10^1^	136,146 ± 29,498^b^	144,015 ± 23,642^b^
1.6 × 10^3^	97,824 ± 21,284^c^	80,630 ± 10,584^c^
1.6 × 10^5^	20,512 ± 7,964^c^	16,656 ± 9,791^c^

Note

Values in the same column with different letters, a, b and c in superscript are statistically different (*p* < .05 and 0.005). The results were expressed as difference in counts per minute (DCPM) and presented as mean ± *SD*.

The effect of AFB_1_ on LPS‐activated SAM was assessed by their uptake of [^3^H] glucosamine. As shown in Table 4, the uptaking values either the DMSO control or AFB_1_‐treated groups were generally low. However, the reaction appeared to be time‐dependent since the values of CPM increased when the incubation period was prolonged from 24 to 48 hr. When SAM were incubated with higher concentrations of AFB_1_, 1.6 × 10^3^ nmol/L (24 hr) and 1.6 × 10^5^ nmol/L (24 and 48 hr), the CPM was significantly (*p* < .05) lower than that of DMSO control. For both incubation periods, none of the tested AFB_1_ concentrations was able to completely block the uptake of [^3^H]‐glucosamine in SAM.

**Table 4:** The toxic effects of different concentrations of aflatoxin B_1_ on the activation of swine alveolar macrophages by LPS and tested by radioactivity assay after 24 and 48 hr of incubation in vitro

Conc. of aflatoxin B_1_ (nmol/L)	Incubation period (hr)	
	24	48
DMSO‐C	294 ± 18^a^	1,030 ± 143^a^
1.6 × 10^–1^	284 ± 34^ab^	894 ± 162^a^
1.6 × 10^1^	262 ± 30^ab^	1,055 ± 127^ab^
1.6 × 10^3^	201 ± 33^bc^	761 ± 68^ab^
1.6 × 10^5^	141 ± 13^c^	655 ± 69^b^

Note

Values in the same column with different letters, a, b and c in superscript are statistically different (*p* < .05 and 0.005). The results were expressed as difference in counts per minute (DCPM) and presented as mean ± *SD*.

## 4 DISCUSSION

This study demonstrated that AFB_1_ had a profound inhibitory effect on physiological functions of SAM in vitro, including phagocytic activity, macromolecular synthesis and LPS‐induced cell activation. Early studies indicated that impairment in the function of the reticuloendothelial system to clear colloidal carbon from the circulation has been reported in chickens fed on a diet containing AFB_1_ at doses as low as 0.125 to 0.3 ppm (Kadian et al., 1988; Michael et al., 1973). Reduced phagocytosis of *A. fumigatus* spores by alveolar macrophages was observed in rabbits given doses of AFB_1_ ranging from 0.03 to 0.09 mg per day for 2 weeks (Richard & Thurston, 1975). Similar inhibitory effects were also found when duck peritoneal macrophages were incubated with 5–100 μg/ml of AFB_1_ for 12 hr (Cheng, Shen, Pang, & Chen, 2002). It was speculated that the reduced phagocytosis was due to decreased formation or activity of complements and other opsonins, and/or possible induction of inhibiting factors in AFB_1_‐treated animals (Richard & Thurston, 1975). A recent study also indicated that naturally occurring levels of AFB_1_ could down‐regulate the key phagocytic element CD64 of human dendritic cells (Mehrzad et al., 2018). The cascade of phagocytosis includes interaction of targets with phagocytic cells followed by target internalization. Since the bacteria used in our study were opsonized with heat‐inactivated serum from normal pigs, the phagocytosis was mainly antibody‐dependent which is mediated by surface Fc receptors. Decrease in the number of Fc receptors could reduce bacterial uptake by SAM. It has been shown that lipid peroxidation induced by oxygen‐derived free radicals decreases the number of surface membrane insulin receptors of rat hepatic cells (Perera, Betschart, Virji, Katyal, & Shinozuka, 1987). A similar study also indicated that AFB_1_ depresses the expression of phenotypic markers of splenic CD8(+) T cells and CD3(−) CD8a(+) NK cells of rats when fed with 5–75 μg of AFB1 for a week (Qian et al., 2014). Our results indicated that AFB_1_ treatment prominently inhibited the protein synthesis in SAM. This inhibition may affect the synthesis of certain crucial proteins involved in phagocytosis, such as actin, myosin and fibronectin besides the formation of Fc receptors (Gerberick et al., 1984), which interfere with the internalization of attached bacteria. Therefore, the reduced phagocytic ability of AFB_1_‐treated SAM in this study may be attributed to decreased interaction between SAM and bacteria, and defective bacterial internalization.

It is known that the broad range of biological effects caused by aflatoxins is, at least partially, related to their reactions with cell nucleic acids and nucleoproteins, so that the protein synthesis, regulation of cell apoptosis and cellular integrity are affected (Applebaum, Brackett, & Wiseman, 1982; Mehrzad et al., 2018; Prasad, Sinha, & Ali, 1997). In this study, we evaluated the effects of AFB_1_ on DNA, RNA and protein synthesis of SAM. All the three macromolecules tested were inhibited in AFB_1_‐treated SAM, in which protein synthesis was affected the most, followed by DNA synthesis, and RNA synthesis was the least sensitive. When SAM were treated with AFB_1_ higher than 1.6 × 10^1^ nmol/L for 24 hr, significant reductions in the synthesis of protein and DNA were observed. Whereas, the inhibitory effect for RNA synthesis was only seen at the highest concentration, 1.6 × 10^5^ nmol/L. The three higher AFB_1_ concentrations, 1.6 × 10^1^, 1.6 × 10^3^ and 1.6 × 10^5^ nmol/L, reduced protein synthesis by 31%, 50% and 90%, respectively, and DNA synthesis by 23%, 32% and 70%, respectively. This suggests that aside from the secondary effects of suppressed DNA and RNA synthesis, AFB_1_ also have negative impacts on the protein synthesis in SAM, which eventually has influence on cytokine secretions, cell apoptosis and even cell death (Mehrzad et al., 2018; Meissonnier et al., 2008; Qian et al., 2014). Inhibited synthesis of these macromolecules, at a sublethal level, by AFB_1_ may alter the functions of SAM and modulate cell‐mediated immune and inflammatory responses upon secondary infections.

Activation is crucial for macrophages and dendritic cells to perform their major functions, such as phagocytosis, regulation of cell apoptosis and production of monokines required for regulating T‐ and B‐cell functions more efficiently (Mehrzad et al., 2018; Meissonnier et al., 2008). It has been demonstrated that activated macrophages preferentially incorporate glucosamine into their cell membrane by means of ultrathin autoradiograph sections and cell fractionation procedures (Hammond & Dvorak, 1972). Our results indicated that incubation with AFB_1_ at higher concentrations, 1.6 × 10^3^ nmol/L and above, for 24 hr could significantly reduce the incorporation of [^3^H]‐glucosamine in SAM. The reduction, however, appeared to be reversible since only SAM incubated with the highest concentration (1.6 × 10^5^ nmol/L) of AFB_1_ for 48 hr had significantly reduced incorporation of [^3^H]‐glucosamine. Moreover, the percentage of reduction dropped from 52% to 36% as compared to the DMSO control. T‐2 mycotoxin has been shown to exhibit a prominent inhibitory effect on the incorporation of labelled glucosamine in rat alveolar macrophages (Gerberick et al., 1984). It was proposed that the suppressive effect of T‐2 mycotoxin on protein synthesis leads to inhibited activation of macrophages, which requires the protein synthesis to be intact (Gerberick et al., 1984). Likewise, inhibited protein synthesis in AFB_1_‐treated SAM may result in reduced activation.

The aflatoxin could be readily absorbed from the site of exposure, usually through the gastrointestinal tract and respiratory tract into blood stream (Agag, 2004; Larsson& Tjalve, 2000). Then, it could get into any organs from its diffusion in the blood stream. Since the SAMs are resident macrophages which are originated from blood monocytes. A long period of exposure to aflatoxin might have direct effects on the monocytes and then the tissue macrophages in all organs including the SAMs. Since macrophages play a crucial role in both non‐specific and specific immune responses. Our results suggest a possible mechanism of AF‐induced adverse effects tested above in SAM could further induced immunosuppression via cell apoptosis, and reduced macrophage‐dependent immunocompetence aside from the depressed humoral and cell‐mediated immunities reported previously (Cysewski et al., 1978; Kadian et al., 1988; Mehrzad et al., 2018; Meissonnier et al., 2008; Neldon‐Oritz & Qureshi, 1992; Panangala et al., 1986). More directly, the AF‐induced functional defects in SAM may increase the pig's susceptibility to other pathogens (Cortinovis, 2013; Creppy, 2002; Cysewski et al., 1978; Park et al., 2015).

## 5 CONCLUSIONS

The direct effects of aflatoxin B_1_ on physiological functions of swine alveolar macrophages were examined in this study. Results demonstrated that a significant reduction in the ability of SAM to uptake bacteria shortly 3 hr after being treated with AFB_1_ at a concentration of 16 nmol/L or above. The effects on the activation of SAM by LPS and synthesis of DNA, RNA and protein synthesis were also significant, among which DNA and protein synthesis were affected the most. In general, most of the effects were dose‐dependent, yet time‐dependent. Finally, we theoretically suggest that exposure of AFB_1_ could have detrimental effects on physiological functions of SAM, which may increase the susceptibility of pigs to various secondary infections.

## AUTHOR CONTRIBUTION

**Victor Fei Pang:** Conceptualization; Data curation; Formal analysis; Funding acquisition; Investigation; Methodology; Project administration; Supervision; Validation; Writing‐original draft; Writing‐review & editing. **Chung‐Feng Chiang:** Data curation; Formal analysis; Methodology; Software. **Chih‐Cheng Chang:** Conceptualization; Data curation; Formal analysis; Methodology; Resources; Software; Writing‐original draft; Writing‐review & editing.
